# Knockdown of long non-coding RNA SNHG8 suppresses the progression of esophageal cancer by regulating miR-1270/BACH1 axis

**DOI:** 10.1080/21655979.2021.2021064

**Published:** 2022-01-22

**Authors:** Yonghong Wu, Yan Liang, Min Li, Haidong Zhang

**Affiliations:** a Department of Medical Insurance and Price, Xiangyang No. 1 People’s Hospital, Hubei University of Medicine, Xiangyang, China; b Hematology Department, Xiangyang No. 1 People’s Hospital, Hubei University of Medicine, Xiangyang, China; c Gastroenterology Department, Xiangyang No. 1 People’s Hospital, Hubei University of Medicine, Xiangyang, China; d Oncology Department, Xiangyang No. 1 People’s Hospital, Hubei University of Medicine, Xiangyang, China

**Keywords:** SNHG8, miR-1270, BACH1, lncRNA, esophageal cancer

## Abstract

The emerging evidence showed that lncRNAs (long non-coding RNAs) could regulate the progression and affect the malignant behaviors of cancers. LncRNA SNHG8 (small nucleolar RNA host gene 8) has been reported to participate in most cancers development. Here in this study, the role of lncRNA SNHG8 in esophageal cancer was uncovered by a series of functional experiments. The expression pattern of SNHG8 in tumor tissues or cells was first detected by qRT-PCR. Using a lentivirus knockdown shRNA is to repress the expression of SNHG8. Subsequently, the *in vitro* and *in vivo* experiments were utilized to evaluate whether the malignant behaviors of esophageal cancer were influenced by knockdown SNHG8. The results indicated that lncRNA SNHG8 should be a cancer-promoting factor with a relatively high expression level in esophageal cancer. Moreover, knockdown SNHG8 inhibited the cell viability and induced cell apoptosis in KYSE30 and TE-1 cells. In addition, based on the results of the binding site analysis and the luciferase reporter system, SNHG8 functions by the miR-1270/BACH1 axis. The follow-up experiments verified that lncRNA SNHG8 could down-regulate the expression of miR-1270 to increase the BACH1 expression. Finally, we confirmed that knockdown SNHG8 retarded the progression of esophageal cancer with a xenograft model. To sum up, our findings suggested that lncRNA SNHG8 is a cancer-promoting factor in esophageal cancer. Knockdown SNHG8 could suppress the progression of esophageal cancer, which implies SNHG8 could be used as a therapeutic target in esophageal cancer.

## Introduction

Esophageal cancer (EC) endangers public health seriously due to its high rates of mortality and morbidity [1,2]. Up to date, the incidence of EC is continually rising and the 5-year survival rate of esophageal cancer patients is less than 30% [3]. In the clinic, two principal pathological types of esophageal cancer are esophageal adenocarcinoma and esophageal squamous cell cancer (ESCC), respectively [4,5]. Among them, ESCC is more common and prevalent, accounting for nearly 70% of cases of esophageal cancer [4,5]. Although a lot of advances have been made for the diagnosis and therapy of esophageal cancer, the prognosis is not satisfactory [4,6]. However, the mechanisms concerned with tumorigenesis and the development of esophageal cancer still need further research.

LncRNAs are a class of RNA gene, which is more than 200bp in transcript length, highly conserved in evolution [7,8]. Unlike the protein-coding genes, lncRNAs perform their biological functions without coding proteins [8,9]. In the usual situation, lncRNAs sponge the cellular miRNAs with the matched binding sites to degrade the combined miRNAs [10,11]. Subsequently, those altered miRNAs lead to changes in downstream expression of specific proteins by binding to the 3ʹUTR (Untranslated Regions) of the target gene to inhibit translation [10]. Additionally, lncRNAs could also be used as a scaffold to prevent engaged protein degradation or influence its localization [12,13]. Thus, lncRNAs have been substantiated to could affect the malignant behaviors of cancer cells to control the progression of cancer [14]. LncRNA SNHG8 (small nucleolar RNA host gene 8) is affiliated with the lncRNA class and associated with most cancers and diseases [15]. Previous studies reported that SNHG8 often plays an oncogenic role in numerous cancers, such as breast cancer, colorectal cancer, and cancer [16–18]. On most occasions, lncRNA SNHG8 exerts its biological functions in a ceRNA (competitive endogenous RNA) manner [15]. For instances, SNHG8 promotes the breast cancer cells proliferation by regulating the miR-335-5p/PYGO2 axis and accelerates ovarian cancer progression by miR-1270/ S100A11 axis [19,20]. Yet, few studies discussed how SNHG8 performs in esophageal cancer. Therefore, the role and functional mechanism of SNHG8 in esophageal cancer need to be further clarified.

Similarly, microRNAs (miRNAs) are another class of RNA gene, which is about 20bp in length [21,22]. MiRNAs could bind the matched sequence of target mRNA, and then it causes mRNA degradation no matter where it binds on 3ʹUTR or other regions [22,23]. MiR-1270 is a representative miRNA, which could regulate the progression of cancer among numerous miRNAs [24]. It has been indicated that miR-1270 usually acts as a tumor suppressor in most cancers [25,26]. In addition, BACH1 (BTB Domain And CNC Homolog 1) encodes a transcription factor to regulate the cell process [27]. Especially in esophageal cancer, BACH1 was considered to be an oncogene promoting the development [28]. However, no studies have linked the miR-1270 and BACH1 together until now. Our study will explore the interactions between SNHG8 and miR-1270 or miR-1270 and BACH1 in esophageal cancer.

To sum up, this study aims at revealing the role of SNHG8 in esophageal. We first proposed that lncRNA SNHG8 participates in regulating the progression of esophageal cancer. The subsequent results verified that knockdown of SNHG8 suppresses the progression of esophageal cancer by modulating the miR-1270/BACH1 axis. Our study provided a novel biomarker for regulating the progression of esophageal cancer.

## Methods

### Tumor tissues

Esophageal cancer tissues and paired adjacent noncancerous tissues were obtained from 20 esophageal cancer patients between 2018 and 2020 with informed written consent. The collected tissues were transferred to a − 80°C refrigerator after quick-frozen in liquid nitrogen. The clinical characteristics of the patients are shown in Table 1.

**Table 1.** The clinical characteristics of the patients

Characteristics	Expression of		P value
	Low (n = 10) High (n = 10)		
Sex			0.3291
Male	8	6	
Female	2	4	
Age			0.3613
≤ 60	3	5	
> 60	7	5	
Lymph node mestasis			0.3711
Yes	4	6	
No	6	4	
Pathological Staging			0.6392
I + II	7	6	
III + IV	3	4	
Distant metastasis			0.3291
M0	8	6	
M1	2	4	
Smoking status			0.6056
Ever/current	8	7	
Never	2	3	
Alcohol consumption			0.6056
Ever/current	8	7	
Never	2	3	

Low/high by the sample mean. Pearson χ2 test. *P < 0.05 was considered statistically significant

### qRT-PCR

Total RNA of corresponding tissues and cells were extracted by Trizol (Invitrogen, Carlsbad, CA, USA) and then reversed to cDNA with a reverse transcription kit (Invitrogen). Quantitative PCR was performed on a CFX96 RT-PCR system (Bio-Rad) using a two-step PCR protocol. In brief, 95°C, 10 min for denaturation, and then enter 40 cycles of 95°C for 15 s and 60°C for 60 s. The relevant primers are listed in Table 2. GAPDH and U6 were, respectively, as internal controls for mRNA and miRNA.

**Table 2.** The primers are as follow

Name	Forward /Reverse	Sequence (5ʹ to 3ʹ)
SNHG8	F	TGGGATAAGGCTGGGTGTAG
	R	TGTCCATCTTTCCCCAAAAG
miR-1270	F	CTGGAGATATGGAAGAGCT
	R	CAGTGCGTGTCGTGGAGT
BACH1	F	TCTGAGTGAGAACTCGGTTTTTG
	R	CGCTGGTCATTAAGGCTGAGTAA
GAPDH	F	GCAACTAGGATGGTGTGGCT
	R	TCCCATTCCCCAGCTCTCATA
U6	F	CCCTTCGGGGACATCCGATA
	R	TTTGTGCGTGTCATCCTTGC

### Cell culture

All cells were obtained from Shanghai Cell Bank of Type Culture Collection of Chinese Academy of Sciences. KYSE30, EC9706, and TE-1 cell lines and human esophageal epithelial cells (Het-1A) were all cultured in RPMI 1640 medium (Gibco, USA) with 10% fetal bovine serum (Gibco, USA) in a 37°C, 5% CO2 incubator.

For cell transfection, lentivirus shRNA (Genechem Shanghai, China, MOI = 25) sh-SNHG8 or sh-NC were to infect EC9706 and KYSE30 cells. After 6 hours, the supernatant was replaced with a medium containing 1 μg/mL puromycin (Sigma, USA) for 2 days to collect the sh-SNHG8 or sh-NC cells. About at 50% confluence, cells were treated with miR-1270 mimic and miR-1270 inhibitor and corresponding negative control (100 pmol/ml, RiBoBio, Guangzhou, China) by Lipofectamine™2000 (ThermoFisher, USA).

### Cell viability

Three thousand cells were plated into a 96-well plate for 24 h (Day 0), 48 h (Day 1), 72 h (Day 2) or 96 h (Day 3) to detect the cell viability. After the indicated time, 10 μl CCK-8 (Beyotime, Shanghai, China) was added into each well for another 4 h. Next, the supernatant was replaced with 150 μl DMSO (Sigma) to examine the results on 450 nm by a Microplate Reader.

### Colony formation

Five hundred cells were planted into a six-well plate for 12 days. During the period, the culture medium was changed according to the condition of the culture medium. On day 12, the cell colony was stained and counted by 1% crystal violet. More than 50 cells were identified as a colony.

### Apoptosis

According to the grouping, cells were harvested at 72 h post-transfection. The apoptosis cells were examined by an Annexin V-FITC/PI kit (Invitrogen, USA) on flow cytometry (Beckman Coulter).

### Luciferase reporter system

Luciferase reporter vectors (pRL-TK, Promega) was to verify the interaction between SNHG8 and miR-1270 or miR-1270 and BACH1. The corresponding plasmids with the wild-type sequence or mutant sequence were synthesized from Genepharma. After 48 h at co-transfection with luciferase plasmids and miR-1270 mimic or miR NC, the intensity was detected by the Dual-Luciferase Reporter Assay System (Promega).

### Western blotting

The cells and tumors are lyzed by RIPA Lysis Buffer (Beyotime, Shanghai, China) to get protein, and then centrifugation and degeneration. The samples were separated by 10% SDS-PAGE gels and transferred to PVDF membrane (Bio-Rad). The primary antibody, anti-BACH1 (1:1000, A5393, ABclonal) and GAPDH (1:5000, ab8245, Abcam) were incubated after blocking with blocking buffer (1% bovine serum albumin (BSA, Sigma-Aldrich)/TBST) overnight. On the second day, HRP conjugated secondary antibodies (1:2000, ab6721 or ab6789, Abcam) were incubated after washing. The results were examined by Chemiluminescence Apparatus (Tanon, 5500).

### The xenograft tumor model

The 10 7-week-old nude female mice were randomly divided into two groups. On day 0, inoculate 6 × 10^6^ sh-NC and sh-SNHG8 EC9706 cells on the flank of nude mice. Every 3 days, record the tumor volume according to volume = 1/2 × length × width^2^. On day 18, we weighted the tumors and then fixed in 4% paraformaldehyde.

### Immunohistochemistry (IHC)

The 4-μm tumor sections were deparaffinized and then incubated with H_2_O_2_ to eliminate endogenous peroxides. After blocking with blocking solution (3% BSA/PBS), the sections were incubated with antibodies, anti-BACH1 (1: 200, A5393, ABclonal) or anti-Ki67 antibody (1:300, ab15580, Abcam) at 4°C overnight. On day 2, the sections were washed to incubate secondary antibody and HRP (Beijing Zhongshan Golden Bridge Biotechnology Co., Ltd). Incubate with 3,3′-diaminobenzidine (DAB, Sigma-Aldrich, St Louis, MO, USA) for 1 min to exhibit the positive marker. IHC images were taken by a light microscope (Olympus).

### Statistical analysis

Statistical analysis was performed using SPSS 19.0 software (SPSS, Chicago, IL, USA). The data are expressed as the mean ± standard deviation (SD). Differences between groups were evaluated by Student’s t-test. P < 0.05 indicated statistical significance.

## Results

This study dedicates to clarify the role of lncRNA SNHG8 in esophageal cancer and how SNHG8 functions in esophageal cancer. Due to its expression is elevated in esophageal cancer compared to normal tissues, we first proposed that lncRNA SNHG8 could participate in regulating the progression of esophageal cancer. To test this conjecture, we knocked the expression level of SNHG8 down in esophageal cancer cells to obverse whether the malignant behaviors are influenced. The subsequent results indicated that knockdown of SNHG8 suppresses the progression of esophageal cancer.

### The aberrant expression of SNHG8 in esophageal cancer tissues and cells

**Figure 1.**
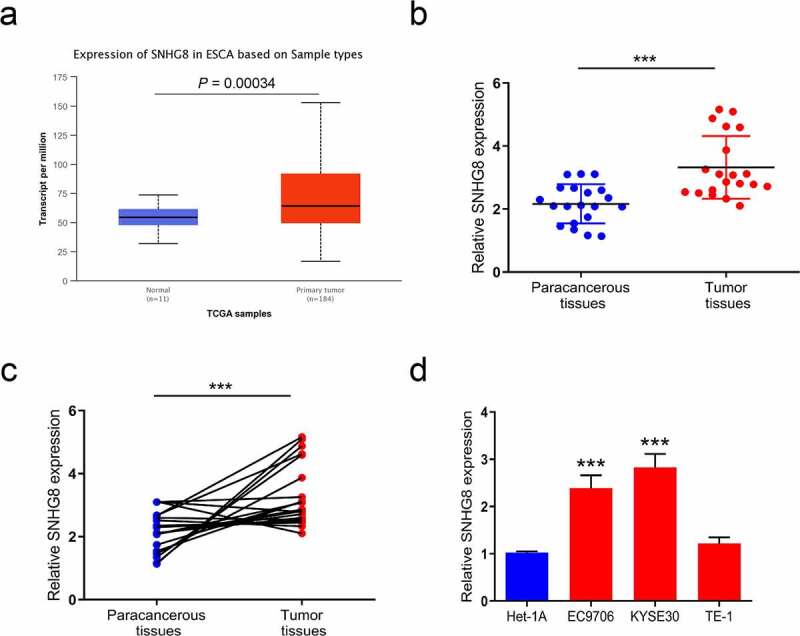
The expression of lncRNA SNHG8 was analyzed in esophageal cancer tissues and cells. (a), The expression levels of SNHG8 in esophageal cancer tissues and normal tissues were analyzed by TCGA database; (b) and (c), The SNHG8 expression in total 20 pairs of esophageal cancer tissues and adjacent tissues was examined by RT-qPCR, N = 20; (d), The levels of SNHG8 were examined by RT-qPCR in Het-1A and esophageal cancer cells, N = 3. *P < 0.05, **P < 0.01, ***P < 0.001.

Most of the genes involved in regulating tumor progression are expressed aberrantly in cancers. First of all, we analyzed the expression pattern of SNHG8 in esophageal cancer by using an online TCGA database (http://ualcan.path.uab.edu/). As the result shown, the expression of SNHG8 in esophageal cancer tissues are significantly enhanced than whose in normal tissues (*P* = 0.00034, Figure 1(a)). Based on this finding, we subsequently detected the expression level of SNHG8 in our collected clinical samples. Consistently, SNHG8 expression was also elevated in esophageal cancer tissues compared to the paired paracarcinoma tissues (Figure 1(b,c)). Meanwhile, the expressions of SNHG8 in esophageal cancer cell lines were examined. In Figure 1(d), compared to the Het-1A cells (Human esophageal epithelial cells), SNHG8 increases in the esophageal cancer cells. Due to the more abundant expression in EC9706 and KYSE30 cells, we chose the two cell lines for the following experiments. In short, we found that lncRNA SNHG8 was elevated in esophageal cancer tissues and cells.

### Knockdown of lncRNA SNHG8 inhibits the proliferation and promotes the apoptosis of esophageal cancer cells

**Figure 2.**
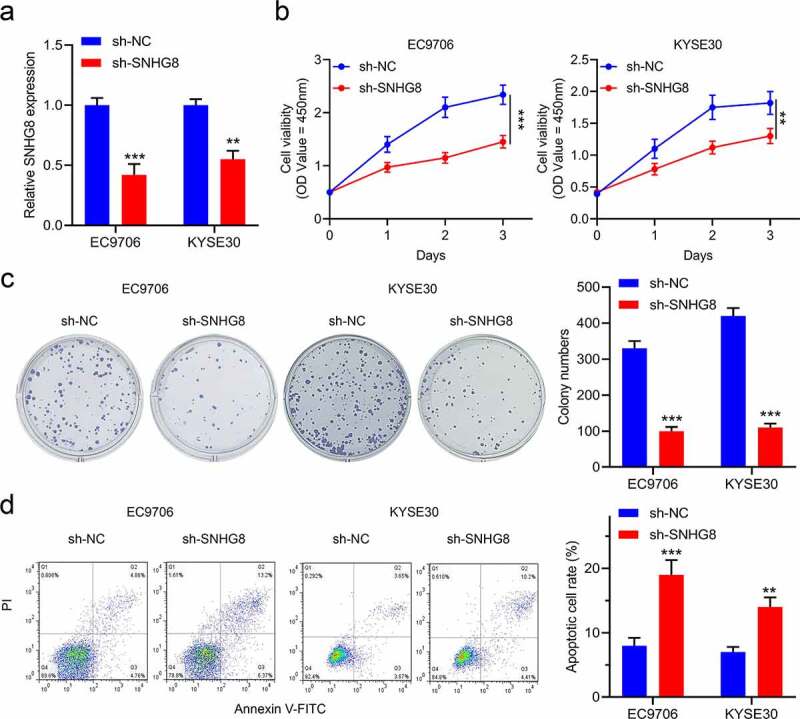
Knockdown of SNHG8 inhibits esophageal cancer cells proliferation and promotes them apoptosis. (a), The expression levels of SNHG8 were detected after cells infected with lentiviral sh-SNHG8 or sh-NC; (b), The cell viability was detected by CCK-8 assay in EC9706 and KYSE30 cells; (c), The colony formation was examined in EC9706 and KYSE30 cells; (d), The apoptosis was examined in EC9706 and KYSE30 cells. N = 3, *P < 0.05, **P < 0.01, ***P < 0.001.

To clarify the role of SNHG8 in esophageal cancer, we constructed a lentivirus shRNA to reduce the expression of SNHG8 (Figure 2(a)). After a successful construction of knockdown SNHG8 in EC9706 and KYSE30 cells, cell viability and apoptotic ability were measured respectively. As shown in Figure 2(b), the cell viability was repressed by knockdown SNHG8 in esophageal cancer cells. Besides it, the colony formation ability of EC9706 and KYSE30 cells receded in sh-SNHG8 cells (Figure 2(c)). In addition, knockdown SNHG8 remarkably increased the apoptosis rate of both EC9706 and KYSE30 cells compared with the sh-NC cells (Figure 2(d)). Thus, knockdown lncRNA SNHG8 inhibits the proliferation and promotes the apoptosis of esophageal cancer cells.

### LncRNA SNHG8 could regulate the expression of BACH1 by reducing miR-1270

**Figure 3.**
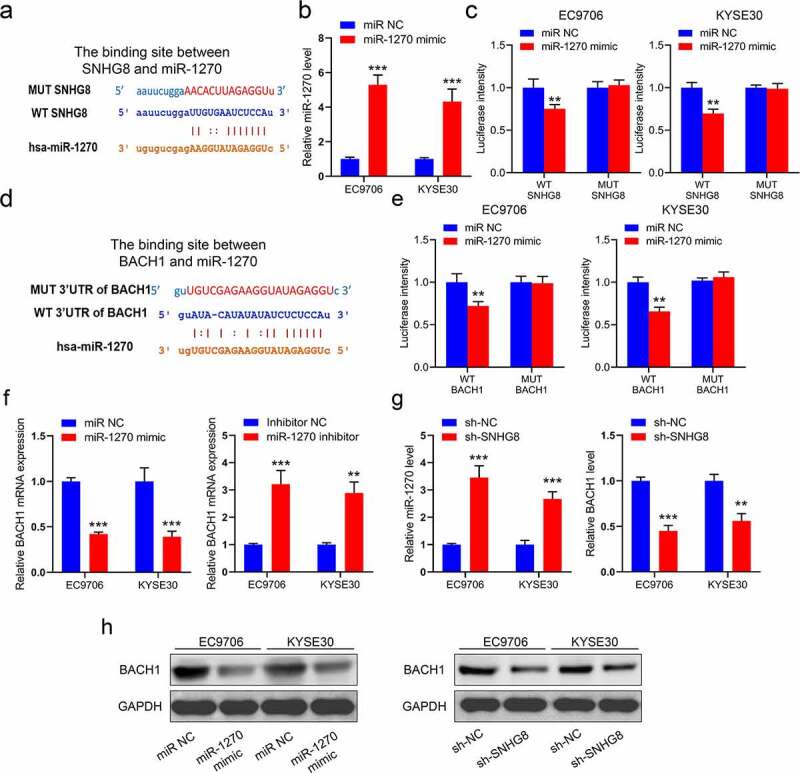
LncRNA SNHG8 could regulate the expression of BACH1 by reducing miR-1270. (a), The binding site between SNHG8 and miR-1270; (b), The expression of miR-1270 in EC9706 and KYSE30 cells with miR-1270 mimic; (c), The luciferase intensity in EC9706 and KYSE30 cells; (d), The binding site between 3ʹUTR of BACH1 and miR-1270; (e), The luciferase intensity in EC9706 and KYSE30 cells; (f), The mRNA expression of BACH1 was detected after transfection; (g), The expression of miR-1270 and BACH1 in sh-SNHG8 EC9706 and KYSE30 cells; (h), The protein expression of BACH1 in EC9706 and KYSE30 cells. N = 3, *P < 0.05, **P < 0.01, ***P < 0.001.

To investigate how SNHG8 exerts its function in esophageal cancer, we first analyzed the downward target of SNHG8. As shown in Figure 3(a), miR-1270 could be a potential target of SNHG8 through conducting an analysis on an online database (https://starbase.sysu.edu.cn/). Therefore, we attempted to confirm the interaction between SNHG8 and miR-1270 by a luciferase reporter system. As the results shown, miR-1270 mimic could increase the miR-1270 expression level in EC9706 and KYSE30 cells (Figure 3(b)) and only the luciferase intensity of wild-type plasmid declined by miR-1270 mimic (Figure 3(c)). Along this line, we further identified the BACH1 is a target of miR-1270 (Figure 3(d)) and miR-1270 mimic only inhibited the luciferase intensity of wild-type 3ʹUTR of BACH1 plasmid, but not mutant type (Figure 3(e)). The mRNA expression of BACH1 was regulated after cells were transfected with miR-1270 mimic or inhibitor (Figure 3(f)). Meanwhile, we found that the expression of miR-1270 augmented and BACH1 level repressed in sh-SNHG8 cells, respectively (Figure 3(g)). As shown in Figure 3(h), the protein level of BACH1 both could be inhibited by miR-1270 mimic or sh-SNHG8. All these results indicated that lncRNA SNHG8 could regulate the expression of BACH1 by reducing miR-1270.

### Inhibition of miR-1270 weakens the knockdown SNHG8 effect on the malignant behaviors of esophageal cancer cells

**Figure 4.**
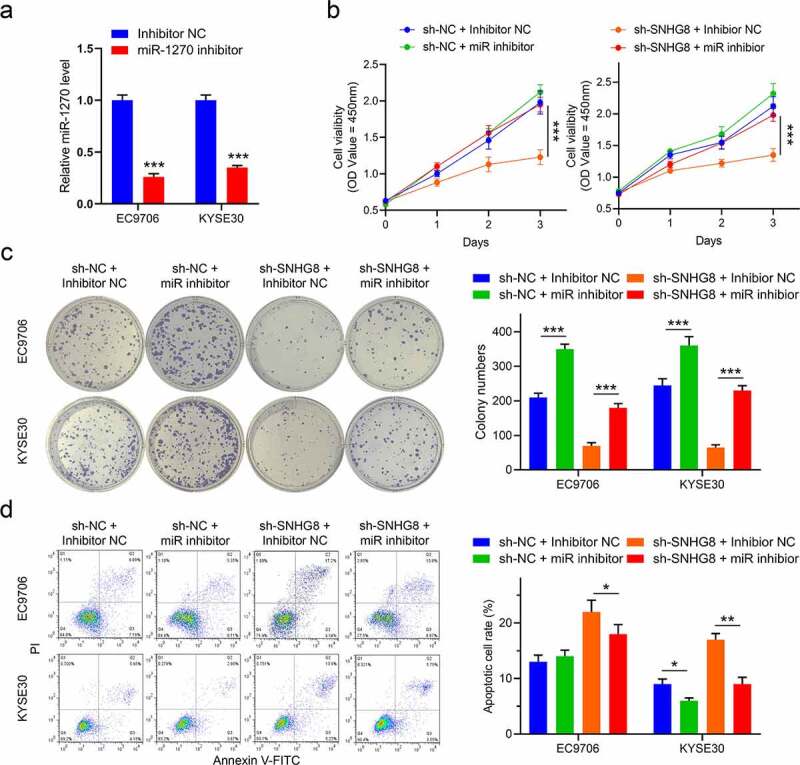
Inhibition of miR-1270 weakens the knockdown SNHG8 effect on the malignant behaviors of esophageal cancer cells. (a), The expression of miR-1270 in EC9706 and KYSE30 cells with miR-1270 inhibitor; (b), The cell viability was detected by CCK-8 assay in EC9706 and KYSE30 cells; (c); The colony formation was examined in EC9706 and KYSE30 cells; (d), The apoptosis was examined in EC9706 and KYSE30 cells. N = 3, *P < 0.05, **P < 0.01.

To prove that lncRNA SNHG8 functions in esophageal by miR-1270/BACH1 axis, we manipulated the related rescue experiments with miR-1270 inhibitor in sh-SNHG8 cells. As shown in Figure 4(a), miR-1270 inhibitor significantly lowered the expression of miR-1270 in esophageal cancer cells. Subsequently, we performed the functional experiments with miR-1270 inhibitor to obverse whether the malignant behaviors were affected. Obviously, miR-1270 inhibitor could weaken the inhibition effect by knockdown SNHG8 on cell viability and colony formation ability of EC9706 and KYSE30 cells (Figure 4(b,c)). Likewise, the apoptosis of esophageal cancer cells got diminish (from 21% to 17% in EC9706 cells; from 18% to 8% in KYSE30 cells) after transfection with miR-1270 inhibitor compared to inhibitor NC (Figure 4(e)). Thus, we revealed that inhibition of miR-1270 weakens the knockdown SNHG8 effect on the malignant behaviors of esophageal cancer cells.

### The xenograft tumor model confirmed that knockdown SNHG8 inhibits the progression of esophageal cancer

**Figure 5.**
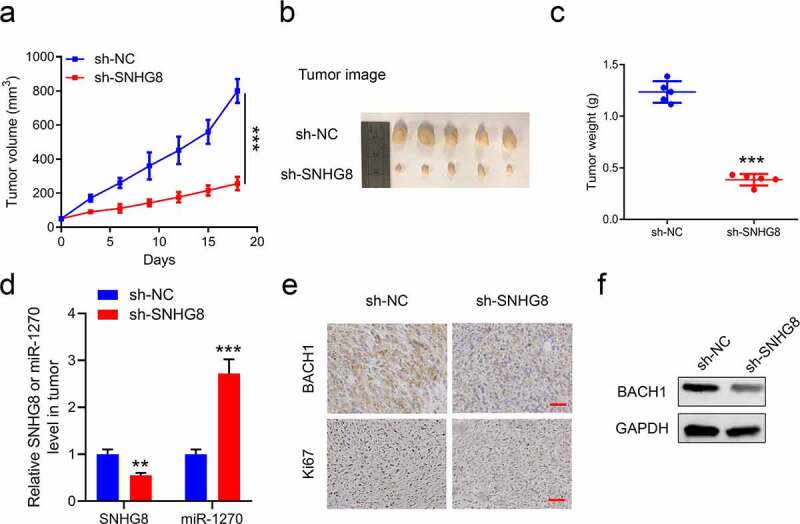
The xenograft tumor model confirmed that knockdown SNHG8 inhibits the progression of esophageal cancer. (a), The tumor volume was recorded every 3 days; (b) The image of tumors; (c), The tumor weight was weighted on Day 18; (d), The levels of SNHG8 and miR-1270 in tumors were examined by qRT-PCR; (e), The BACH1 protein level and Ki67 level were detected by IHC in tumors, Scale bar: 20 and 50 µm, respectively; (f), The BACH1 protein expression was confirmed by WB in tumors. N = 5, **P < 0.01, ***P < 0,001.

Finally, the xenograft tumor model was used to confirm that knockdown SNHG8 inhibits the progression of esophageal cancer. The sh-SNHG8 EC9706 cells were established to an *in vivo* model. During the 18 days, the tumor volume was recorded (Figure 5(a)). On day 18, the tumors were stripped to photo and weight (Figure 5(b,c)). These results showed that the tumor growth was suppressed in sh-SNHG8 cells. Through a further analysis, it uncovered that the expression levels of SNHG8 and miR-1270 were, respectively, descending and ascending in tumors (Figure 5(d)). Moreover, the ki67 rate, which represents the tumor proliferation, also declined in sh-SNHG8 tumors (Figure 5(e)). In addition, the BACH1 level was decreased by knockdown SNHG8 in tumors. (Figure 5(e,f)). All in all, our data suggested that knockdown SNHG8 inhibits the progression of esophageal cancer.

## Discussion

In our study, we revealed that lncRNA SNHG8 should be an oncogene participating in regulating the progression of esophageal cancer. By a lentivirus shRNA, the esophageal cancer cells viability occurred an obvious inhibition effect and the cell apoptosis of esophageal cancer cells was induced after knockdown of the SNHG8, respectively. Moreover, the expression levels of miR-1270 and BACH1 were affected as the expression of SNHG8 changed. Then, we found that miR-1270 inhibitor could weaken the inhibition effect of shSNHG8 on esophageal cancer cells behavior. Thus, we concluded that knockdown SNHG8 suppresses the progression of esophageal cancer by modulating the miR-1270/BACH1 axis.

It has been basically agreed that SNHG8 can promote the progression of cancer [15,17,29]. For instance, in breast cancer, SNHG8 accelerates cell migration and proliferation by miR-335-5p and PYGO2 [16]. At the same time, Xu X et. al. also verified that SNHG8 modulates miR-634/ZBTB20 axis to promote breast cancer progression [29]. In addition, a large number of studies indicated that SNHG8 serves as a cancer-promoter in ovarian cancer, prostate cancer, and nasopharyngeal carcinoma [20,30,31]. To summarize, our findings are agreed with the numerous reports that SNHG8 should be an oncogene participating in regulating the progression of esophageal cancer. In addition, the role of lncRNA SNHG8 was uncovered, the function of miR-1270 and BACH1 was mentioned as well. According to our mechanism, miR-1270 should be a tumor suppressor and BACH1 could promote cancer development in esophageal cancer, respectively. Consistently, miR-1270 was revealed to inhibit human glioblastoma cancer malignant behavior, which played an anti-tumor effect [24]. Literature also suggests that miR-1270 indirectly exhibited a tumor-suppressive outcome in several cancers [25,26]. Moreover, BACH1 has been considered to promote the development of esophageal cancer [28]. Not only that, multiple pieces of research pointed out that BACH1 has been a potential therapeutic target for most cancers [27,32]. A review about BACH1 clarified that BACH1 is closely related to carcinogenic associated factors such as apoptosis, cell cycle, angiogenesis, and oxidative stress [27]. Taken together, our findings are in accordance with the existing literature results. This further increases the reliability of our results.

Nevertheless, some unaccomplished deeper research needs to be further investigated. Although we have identified the anticancer role of SNHG8 in esophageal cancer, there could still be other mechanisms of how SNHG8 regulates cancer progression. May the other downward target miRNAs could be influenced by SNHG8 eventually affect the other downward target protein expression. In addition, the BACH1 is a transcription factor that regulates several signaling pathways [33]. Whether the related with the progression of cancer signaling pathway would be induced or inhibited is need to further investigated. Last but not least, how to combine SNHG8 with tumor therapy such as radiotherapy, chemotherapy, immunotherapy, even and surgical treatment also needs to be developed.

## Conclusion

In this study, we verified that lncRNA SNHG8 participates in regulating the progression of esophageal cancer. Knockdown of SNHG8 suppresses the progression of esophageal cancer by modulating the miR-1270/BACH1 axis.

## Abbreviations

EC: esophageal cancer; ESCC: esophageal squamous cell cancer; lncRNAs: long non-coding RNAs; ceRNA: competing endogenous RNA; SNHG8: small nucleolar RNA host gene 8; miRNAs: microRNAs; 3ʹUTR: 3ʹ Untranslated Regions; BACH1: BTB Domain And CNC Homolog 1; IHC: immunohistochemistry.
